# Supplementary material for: Abnormal Liver Function Tests and Long-Term Outcomes in Patients Discharged after Acute Heart Failure
Source: J Clin Med. 2021 Apr 16;10(8):1730. doi: 10.3390/jcm10081730 (PMC8072793; doi:10.3390/jcm10081730)
Supplement: Supplementary file 1 [file jcm-10-01730-s001.zip › jcm-1171037-supplementary.pdf]

SUPPLEMENTAL MATERIALS

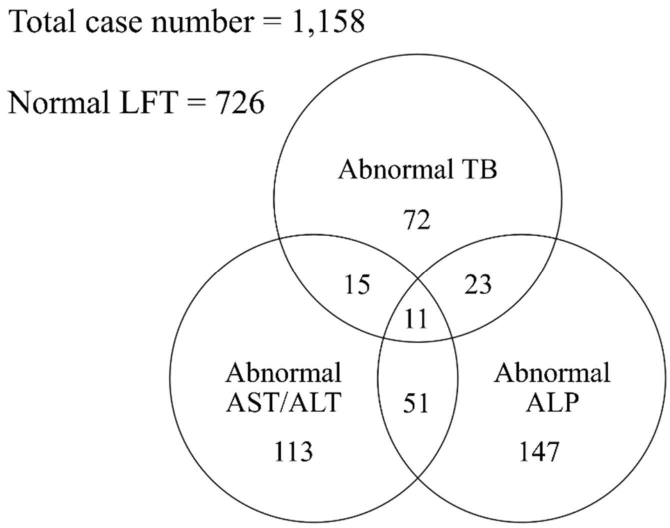

Figure S1. Diagram of number of abnormal liver functions tests. Abbreviations: LFT = liver function test; TB = total bilirubin; AST = aspartate aminotransferase; ALT = alanine aminotransferase; ALP = alkaline phosphatase.

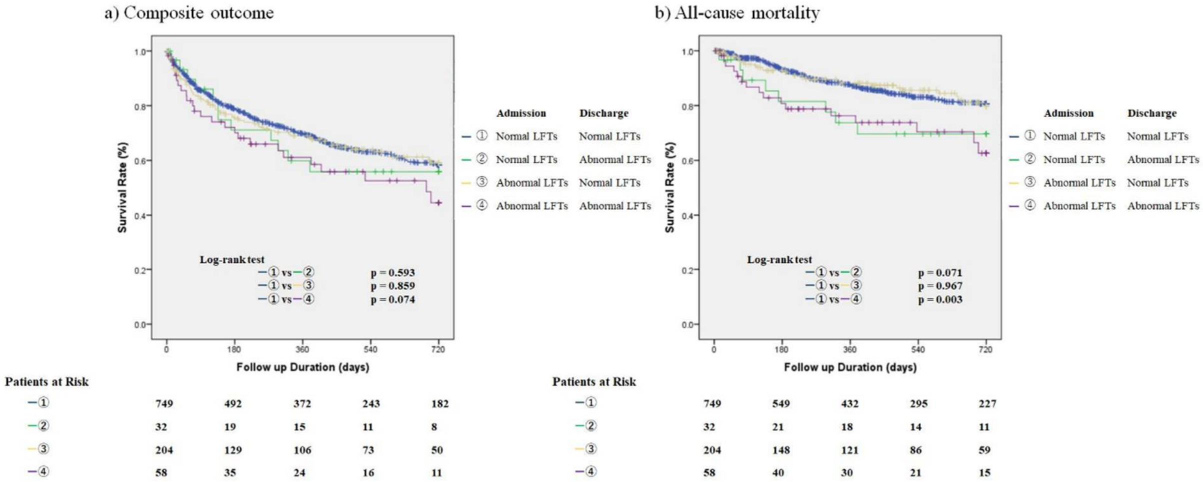

Figure S2. The change in LFTs during hospitalization and patient outcomes. Abbreviations: LFT = liver function test.

Table S1. Comparison of included and excluded patients

Variables	Included Patients N=1,158	Excluded Patients N=1,312	P value
Age, years	77 (66－84)	78 (70－85)	0.001
Male, %	58.4	58.2	0.97
BMI, kg/m^2	23.0 (20.5－26.0)	23.1 (20.6－25.9)	0.86
SBP at discharge, mm Hg	110 (100－122)	110 (100－122)	0.18
HR at discharge, beat per minute	71 (62－80)	70 (62－80)	0.11
NYHA class at discharge			<0.001
Class I or II, %	87.2	74.4	
Class III, %	11.5	23.3	
Class IV, %	1.3	2.3	
LVEF, %	45.0 (31.0－58.0)	47.0 (33.0－59.0)	0.077
Ischemic etiology, %	28.4	30.6	0.13
Comorbidities			
Prior admissions for heart failure, %	26.0	25.3	0.71
Hypertension, %	65.5	68.1	0.18
Hyperlipidemia, %	39.4	39.4	1.00
Diabetes, %	32.0	34.5	0.22
Atrial fibrillation, %	47.7	49.2	0.44
Stroke, %	15.3	13.4	0.19
COPD, %	4.4	4.8	0.63
Hemodialysis, %	2.1	3.5	0.038
Laboratory findings at discharge			
Haemoglobin, mg/dL	11.9 (10.4－13.5)	11.9 (10.5－13.3)	0.87
Sodium, mEq/L	139 (137－141)	139 (137－141)	0.72

Variables	Included Patients N=1,158	Excluded Patients N=1,312	P value
Cr, mg/dL	1.01 (0.80−1.38)	1.01 (0.79−1.38)	0.97
BUN, mg/dL	22.0 (16.3−30.8)	21.8 (16.1−31.7)	0.64
GFR, mL/min/1.73m^2	50.9 (34.8−65.5)	50.3 (35.1−63.8)	0.44
TB, mg/dL	0.7 (0.5−1.0)	0.7 (0.5−1.0)	0.15
AST, IU/L	23.0 (19.0−31.0)	23.0 (18.0−31.0)	0.42
ALT, IU/L	18.0 (12.0−29.0)	17.0 (11.0−27.0)	0.14
ALP, IU/L	232 (187−297)	224 (181−286)	0.17
Albumin, g/dL	3.5 (3.2−3.8)	3.5 (3.1−3.8)	0.25
BNP, pg/mL	266 (134−518)	275 (138−500)	0.82
Prescription at discharge			
Diuretics, %	72.0	75.6	0.048
RAS inhibitor, %	58.4	61.2	0.16
Beta blocker, %	77.7	74.6	0.073
OAC, %	57.4	54.5	0.16

Values are median [interquartile range]. Abbreviations: BMI = body mass index; SBP = systolic blood pressure; HR = heart rate; NYHA = New York Heart Association; LVEF = left ventricular ejection fraction; COPD = chronic obstructive pulmonary disease; BUN = blood urea nitrogen; eGFR = estimated glomerular filtration rate; TB = total bilirubin; AST = aspartate aminotransferase; ALT = alanine aminotransferase; ALP = alkaline phosphatase; BNP = B-type natriuretic peptide; RAS = renin-angiotensin system; OAC = oral anticoagulant.

Table S2. Physical examinations and liver function tests at admission

Variables	
Physical examinations at admission	
PND, %	43.3
Orthopnea, %	57.4
S3 gallop, %	36.1
JVD, %	55.3
Peripheral edema, %	69.0
Cold extremities, %	26.1
Low pulse pressure (less than 40mmHg), %	17.5
Liver function tests at admission	
TB, mg/dL	0.9 (0.6−1.3)
AST, IU/L	34.0 (23.0−49.0)
ALT, IU/L	23.0 (14.0−39.0)
ALP, IU/L	260 (204−340)

Values are median [interquartile range]. Abbreviations: PND = paroxysmal nocturnal dyspnea; JVD = jugular vein distension; TB = total bilirubin; AST = aspartate aminotransferase; ALT = alanine aminotransferase; ALP = alkaline phosphatase.

Table S3. Relationship between LFTs at admission and physical examinations

	OR (95%CI), P value						
	**PND**	**orthopnoea**	**S3 gallop**	**JVD**	**peripheral oedema**	**cold extremities**	**pulse pressure<40**
abnormal TB	1.54 (1.14-2.08)<br>P = 0.005	1.27 (0.93-1.74)<br>P = 0.14	1.17 (0.86-1.58)<br>P = 0.33	1.76 (1.29-2.41)<br>P < 0.001	1.43 (1.03-1.98)<br>P = 0.034	1.00 (0.69-1.46)<br>P = 0.99	1.94 (1.42-2.65)<br>P < 0.001
abnormal AST/ALT	1.31 (1.02-1.68)<br>P = 0.034	1.47 (1.14-1.91)<br>P = 0.004	1.21 (0.94-1.56)<br>P = 0.14	1.53 (1.19-1.98)<br>P = 0.001	0.94 (0.72-1.21)<br>P = 0.61	1.52 (1.11-2.08)<br>P = 0.009	1.83 (1.40-2.41)<br>P < 0.001
abnormal ALP	0.89 (0.67-1.17)<br>P = 0.40	1.11 (0.83-1.49)<br>P = 0.48	0.98 (0.74-1.31)<br>P = 0.90	1.08 (0.81-1.44)<br>P = 0.60	1.26 (0.93-1.69)<br>P = 0.13	1.59 (1.13-2.23)<br>P = 0.007	1.16 (0.85-1.57)<br>P = 0.35
Combined elevation of LFT	1.37 (1.02-1.84)<br>P = 0.038	1.46 (1.06-2.00)<br>P = 0.020	1.23 (0.92-1.66)<br>P = 0.17	1.71 (1.25-2.33)<br>P = 0.001	1.18 (0.87-1.62)<br>P = 0.29	1.51 (1.06-2.14)<br>P = 0.023	1.68 (1.23-2.29)<br>P = 0.001

Supplemental Table 3 shows the logistic regression analysis to assess the association between liver function disorder and physical examination findings after adjustment for sex and age. While the associations between each single abnormality of the LFT panels and physical examination

findings of HF were limited, the combined elevation of LFTs was significantly associated with physical findings reflecting both congestion (i.e. PND, orthopnoea, and JVD) and tissue hypoperfusion (i.e. cold extremities and low pulse pressure). Abbreviations: LFT = liver function test; TB = total bilirubin; AST = aspartate aminotransferase; ALT = alanine aminotransferase; ALP = alkaline phosphatase; PND = paroxysmal nocturnal dyspnoea; JVD = jugular vein distension.
